# Rationale and design of the “Tocilizumab in patients with moderate to severe COVID-19: an open-label multicentre randomized controlled” trial (TOCIBRAS)

**DOI:** 10.5935/0103-507X.20200060

**Published:** 2020

**Authors:** Danielle Leão Cordeiro de Farias, João Prats, Alexandre Biasi Cavalcanti, Regis Goulart Rosa, Flávia Ribeiro Machado, Otávio Berwanger, Luciano César Pontes de Azevedo, Renato Delascio Lopes, Álvaro Avezum, Leticia Kawano-Dourado, Lucas Petri Damiani, Salomón Soriano Ordinola Rojas, Cleyton Zanardo de Oliveira, Luis Eduardo Coelho Andrade, Alex Freire Sandes, Maria Carolina Pintão, Claudio Galvão de Castro Júnior, Phillip Scheinberg, Viviane Cordeiro Veiga

**Affiliations:** 1 BP - A Beneficência Portuguesa de São Paulo - São Paulo (SP), Brazil.; 2 Brazilian Research in Intensive Care Network (BRICNet) - São Paulo (SP), Brazil.; 3 Research Institute, HCor-Hospital do Coração - São Paulo (SP), Brazil.; 4 Hospital Moinhos de Vento - Porto Alegre (RS), Brazil.; 5 Academic Research Organization, Hospital Israelita Albert Einstein - São Paulo (SP), Brazil.; 6 Hospital Sírio-Libanês - São Paulo (SP), Brazil.; 7 Duke Clinical Research Institute, Duke University Medical Center, Durham, NC, Estados Unidos.; 8 Brazilian Clinical Research Institute - São Paulo (SP), Brazil.; 9 International Research Center, Hospital Alemão Oswaldo Cruz - São Paulo (SP), Brazil.; 10 Fleury Medicina e Laboratórios - São Paulo (SP), Brazil.; 11 Santa Casa de Porto Alegre - Porto Alegre (RS), Brazil.

**Keywords:** Coronavirus, Tocilizumab, Covid-19, SARS-CoV-2, Coronavírus, Tocilizumabe, Covid-19, SARS-COV-2

## Abstract

**Introduction:**

Pro-inflammatory markers play a significant role in the disease severity of patients with COVID-19. Thus, anti-inflammatory therapies are attractive agents for potentially combating the uncontrolled inflammatory cascade in these patients. We designed a trial testing tocilizumab versus standard of care intending to improve the outcomes by inhibiting interleukin-6, an important inflammatory mediator in COVID-19.

**Methods and analysis:**

This open-label multicentre randomized controlled trial will compare clinical outcomes of tocilizumab plus standard of care versus standard of care alone in patients with moderate to severe COVID-19. Two of the following four criteria are required for protocol enrolment: D-dimer > 1,000ng/mL; C reactive protein > 5mg/dL, ferritin > 300mg/dL, and lactate dehydrogenase > upper limit of normal. The primary objective will be to compare the clinical status on day 15, as measured by a 7-point ordinal scale applied in COVID-19 trials worldwide. The primary endpoint will be assessed by an ordinal logistic regression assuming proportional odds ratios adjusted for stratification variables (age and sex).

**Ethics and dissemination:**

The TOCIBRAS protocol was approved by local and central (national) ethical committees in Brazil following current national and international guidelines/directives. Each participating center had the study protocol approved by their institutional review boards before initiating protocol enrolment. The data derived from this trial will be published regardless of the results. If proven active, this strategy could alleviate the consequences of the inflammatory response in COVID-19 patients and improve their clinical outcomes.

## Abstract (pt)

**Introdução:**

Os marcadores pró-inflamatórios desempenham papel importante na severidade de pacientes com COVID-19. Assim, terapêuticas anti-inflamatórias são agentes interessantes para potencialmente combater a cascata inflamatória descontrolada em tais pacientes. Delineamos um ensaio para testar tocilizumabe em comparação com o tratamento padrão, tendo como objetivo melhorar os desfechos por meio da inibição da interleucina 6, um importante mediador inflamatório na COVID-19.

**Métodos e análises:**

Este será um estudo aberto multicêntrico, randomizado e controlado, que comparará os desfechos de pacientes tratados com tocilizumabe mais tratamento padrão com o tratamento padrão isoladamente em pacientes com COVID-19 moderada a grave. Como critérios de inclusão, serão exigidos dois dos quatro critérios a seguir: dosagens de dímero D acima de 1.000ng/mL, proteína C-reativa acima de 5mg/dL, ferritina acima de 300mg/dL e desidrogenase lática acima do limite superior do normal. O objetivo primário será comparar a condição clínica no dia 15, conforme avaliação por meio de escala ordinal de 7 pontos aplicada nos estudos de COVID-19 em todo o mundo. O desfecho primário será avaliado por regressão logística ordinal assumindo razões de propensão proporcionais ajustadas pelas variáveis de estratificação (idade e sexo).

**Ética e disseminação:**

O TOCIBRAS foi aprovado pelos comitês de ética locais e central (nacional) do Brasil em conformidade com as atuais diretrizes e orientações nacionais e internacionais. Cada centro participante obteve aprovação do estudo por parte de seu comitê de ética em pesquisa, antes de iniciar as inscrições no protocolo. Os dados derivados deste ensaio serão publicados independentemente de seus resultados. Se tiver sua efetividade comprovada, esta estratégia terapêutica poderá aliviar as consequências da resposta inflamatória na COVID-19 e melhorar os resultados clínicos.

## INTRODUCTION

At the end of 2019, a new coronavirus outbreak in Wuhan (China) made headlines as cases of severe pneumonia and deaths began to emerge.^(1)^ The new virus was first named 2019-nCoV, and little was known about its pathogenesis, transmissibility, and lethality rates.^(1,2)^ Cases started to spread within China and in 2020 beyond its borders to all continents (except Antarctica). In February 2020, the World Health Organization (WHO) adopted the official name SARS-CoV-2 (instead of 2019-nCoV), and the syndrome associated with the new coronavirus infection was named COVID-19, and declared a pandemic in mid-March.^(3)^ By June 2020, almost 9 million cases and 470 thousand deaths have been reported worldwide.^(4)^ In Brazil, the first reported cases were in late February, and by June, the country accumulates more than one million cases and 50,000 deaths.

The development of pneumonia progressing to a systemic inflammatory response syndrome (SIRS) and multiorgan failure indicates that pro-inflammatory cytokines are a significant contributor to morbidity and mortality in SARS-CoV-2 infection.^(5,6)^ The rapid rise in cytokines reminiscent of that seen in chimeric antigen receptor therapy (CAR-T) is proposed to be an essential element in the rapid clinical deterioration.^(7-9)^ The COVID-19 SIRS is usually observed in the second week of infection, where a slew of pro-inflammatory signals are triggered, leading to respiratory compromise, and multiorgan failure. After SARS-CoV-2 initially binds to the angiotensin-converting enzyme 2 (ACE2) receptor, several cell types are infected thereafter from the respiratory, gastrointestinal, neurologic, endothelial, and reticuloendothelial systems, giving rise to a wide clinical presentation ranging from oligosymptomatic to multiorgan failure and death.^(10-15)^ Clinical and laboratory risk factors associated with worse outcomes are being recognized as the pandemic unfolds globally.^(16-18)^

Macrophage infection and activation, initially in the lungs and then systemic, emerges as an essential source of pro-inflammatory cytokines such as interferon gamma (IFN-γ), interleukin (IL)-6, IL-12, tumoral necrosis factor (TNF), IL-1RA, and C-X-C motif chemokine 10 (CXCL10) among others.^(5)^ Other systems, such as the coagulation cascade, are activated by the crescent inflammatory response, leading to thrombotic and hemorrhagic events both in the lung and systemically.^(19,20)^ This multisystem havoc triggered by SARS-CoV-2 is now recognized to be a critical element to its pathogenesis; this rationale has guided the proposal of therapeutic strategies that could possibly block its more lethal consequences. For example, an old drug, etoposide, and several more targeted approaches emerged in response to the macrophage activation, including anti-cytokine, anti-chemokine, anti-complement therapies, and Janus kinase (JAK) and Bruton tyrosine kinase (BTK) inhibitors.^(8,21)^ It is likely that, if proven active, the benefit of these therapies will be incremental, and a combination strategy with antivirals, anticoagulants, or possibly convalescent plasma, will provide a better therapeutic approach.^(22-24)^

Tocilizumab is an IL-6 inhibitor approved for rheumatoid arthritis, giant cell arteritis, and cytokine release syndrome during CAR-T.^(25,26)^ Preliminary data have shown that tocilizumab and other IL-6 blockers may have a role in severe SIRS cases, but its role in COVID-19 patients has not yet been confirmed, and its use should be considered experimetal.^(27,28)^ Given its mode of action, it is plausible that tocilizumab is active in patients with already established SIRS (that could worsen), as opposed to the early use during the infection cycle with no demonstrable inflammatory activity. Thus, to test this hypothesis, we devised a trial where tocilizumab will be tested to avert the more lethal consequences of COVID-19-related SIRS. To investigate the effects of blocking an ongoing inflammatory response, we designed a randomized controlled trial comparing the efficacy of tocilizumab plus standard of care to standard of care alone in patients with moderate to severe SARS-CoV-2 infection who require supplemental oxygen and have increased systemic inflammation markers levels. The requirement of having increased levels of systemic inflammatory markers, in our hypothesis, will better select those more likely to benefit from this strategy.

## METHODS

### Study design

**Figure 1:**
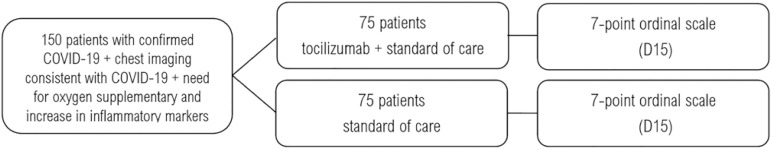
Schedule of enrollment, interventions, and assessments. D - day.

The TOCIBRAS (Tocilizumab in patients with moderate to severe COVID-19: an open-label multicentre randomized controlled” trial) was developed according to SPIRIT guidelines (Appendix 1) and registered in clinicaltrials.gov as NCT04403685. The TOCIBRAS trial is an open-label, parallel-group, superiority, multicenter, randomized controlled trial with the primary objective to show that tocilizumab, added to the standard of care, is superior to the standard of care alone in moderate to severe cases of COVID-19 (Figure 1).

This study is being conducted by the Coalition COVID-19 Brazil, a collaborative research network including the following hospitals: *BP - A Beneficência Portuguesa de São Paulo*, *HCor-Hospital do Coração*, *Hospital Israelita Albert Einstein*, *Hospital Sírio-Libanes*, *Hospital Alemão Oswaldo Cruz*, *Hospital Moinhos de Vento*, Brazilian Clinical Research Institute (BCRI), and the Brazilian Research in Intensive Care Network (BRICNet) (Appendix 2).

### Eligibility criteria

#### Inclusion criteria

1. Confirmed diagnosis of SARS-CoV-2 infection.
2. Chest computed tomography (or chest X-ray) consistent with COVID-19.
3. COVID-19-related symptoms for more than three days.
4. 18 years of age or older.
5. Need for oxygen supplementation to maintain oxygen saturation (SpO_2_) > 93% OR need for mechanical ventilation less than 24 hours before the randomization.

6. Two or more of the following inflammatory tests:
  - D-dimer > 1,000ng/mL.
  - C reactive protein (CRP) > 5mg/dL.
  - Ferritin > 300mg/dL.
  - Lactate dehydrogenase (LDH) > upper limit of normal.

#### Exclusion criteria

1. Need for mechanical ventilation for 24 hours or more before the randomization
2. Hypersensitivity to tocilizumab
3. Patients without therapeutic perspective or in palliative care
4. Active non-controlled infections (other than COVID-19)
5. Neutrophil count < 0.5 x 109/L
6. Platelet count < 50 x 109/L
7. Liver disease, cirrhosis or elevated aspartate aminotransferase (AST) or alanine aminotransferase (ALT) above 5 times the upper limit of normal.
8. Renal disease with estimate glomerular filtration below 30mL/min/1.72 m2 (Modification of Diet in Renal Disease - MDRD or Chronic Kidney Disease Epidemiology Collaboration - CKD-EPI scores).
9. Breastfeeding women.
10. Pregnancy.
11. Other clinical conditions that contraindicate tocilizumab, according to the attending physician.

### Outcomes

#### Primary objective

To assess the effect of tocilizumab plus standard of care compared to standard of care alone on the clinical status on day 15, as measured by a 7-point ordinal scale, in adults hospitalized patients with moderate to severe COVID-19. The 7-point ordinal scale applied in this study is as follows:

1. Patient not hospitalized, with no limitation in activities.
2. Patient not hospitalized, with limitation in activities.
3. Patient in the hospital, without supplemental oxygen.
4. Patient in the hospital, with supplemental oxygen.
5. Patient in the hospital on non-invasive positive pressure ventilation (NIPPV) or high flow nasal cannula.
6. Patient on mechanical ventilation.
7. Death.

#### Secondary objectives

To assess the treatment effect with tocilizumab plus standard of care compared to standard of care, in adults hospitalized with COVID-19, on the following outcomes:

1. All-cause mortality from randomization until day 28.
2. Hospital mortality.
3. Degree of organ dysfunction assessed with the Sequential Organ Failure Assessment (SOFA) score on day 8 and day 15 after randomization.
4. Clinical status on days 8 and 29 after randomization, using the 7-level ordinal scale.
5. Ventilator-free days within 29 days.
6. Time until oxygen support independence within 29 days.
7. Length of hospital stay.
8. Incidence of secondary infections.
9. Thromboembolic events (stroke, myocardial infarction, deep vein thrombosis, pulmonary thromboembolism).
10. Incidence of adverse events (AE).

### Exploratory secondary objectives

1. To assess the inflammatory markers and cytokines’ association with the clinical outcomes.
2. To assess the kinetics of hemostatic parameters, inflammatory markers, cytokines, peripheral blood flow cytometry, complete blood count, renal and liver function tests.
3. To assess the viral clearance of SARS-CoV2 on D8.

The following exploratory tests will be conducted and correlated with clinical outcomes:

- Biomarker measurements: D-dimer, CRP, LDH, ferritin, IL-6, TNFα, IL2 receptor (CD25), and IL-10.
- Peripheral blood cytometry for T (CD4+, CD8+, double-negative T cells subpopulations), B (transitional, naïve, non-class switched memory cells, class-switched memory cells, and plasma cells) and NK (CD16+/CD56-, CD16+/CD56+, CD16-/CD56++) lymphocytes, monocytes (classical, intermediate and non-classical), plasmacytoid and myeloid dendritic cells, eosinophils, basophils, and neutrophils.
- Coagulation studies PT/PTT, fibrinogen, vWF, ristocetin cofactor, and factor 8.

Details of the methodology for all these tests is provided in appendix 3. 

### Interventions

The concomitant use of hydroxychloroquine, azithromycin, corticosteroids, or other therapies are allowed in this trial as part of the standard of care if local institutional guidelines/protocols include these agents as part of the standard management for COVID-19 patients. Antibiotics are permitted at any time during the study at the discretion of the treating physician. The standard of care treatment for COVID-19 is not yet defined thus investigators can apply what is considered their standard approach for these patients per local policies. 

After providing written informed consent, the eligible patients will be randomized to receive either tocilizumab plus standard of care (n = 75) or standard of care alone (n = 75). In the experimental arm, tocilizumab will be given as a single intravenous infusion at 8 mg/kg dose. The maximum dose is 800 mg. To allow for homogenous dose rounding between centers, the following bodyweight-based scale will be applied:

- < 50kg - 8mg/kg/dose.
- From 50 to 56kg - 400mg/dose.
- From 57 to 68kg - 500mg/dose.
- From 69 to 81kg - 600mg/dose.
- From 82 to 93kg - 700mg/dose.
- ≥ 94kg to 800mg/dose (max dose).

No dose adjustment for renal or hepatic impairment will be used. Infusion-related reactions will be treated with antihistamines and corticosteroids as needed and reported as AE. All infusions will be administered in hospitalized patients in a regular ward or intensive care unit. 

### Randomization

The participants will be randomly assigned to either the experimental (tocilizumab) or the control (standard of care) group with a 1:1 allocation considering blocks (2, 4, 6, and 8) with random variation and using age (< 60 and ≥ 60 years) and gender as strata, according to a computer-generated randomization schedule. The random sequence will be generated by a statistician not involved with patient’s care using a Software R 3.6.3 algorithm.^(29)^

Allocation concealment is ensured by a web-accessed system (REDCap) which only displays the assigned treatment after the participant is properly registered into the trial system and all eligibility criteria are met.^(30,31)^

### Blinding

Patients, investigators, and caregivers will not be blinded to the assigned treatment. Clinical outcome assessors and statisticians performing the analysis are not involved with the patient care teams and are independent of the treating sites.

### Data collection, management, analysis

Study data will be collected and managed using Research Electronic Data Capture (REDCap) electronic data capture tools hosted at *BP - A Beneficência Portuguesa de São Paulo*.^(30,31)^REDCap is a secure, web-based software platform designed to support data capture for research studies, providing an intuitive interface for validated data capture; audit trails for tracking data manipulation and export procedures; automated export procedures for seamless data downloads to common statistical packages and procedures for data integration and interoperability with external sources.

Data will be entered by the local study personnel who have a unique center designated access, which is not transferable. A limited number of staff members are provided access to REDCap for study-related interactions.The data will be kept on the institutional server, under the recommended REDCap Consortium security requirements.^(31)^ This system’s functionalities include patient registration, concealment of randomization, data entry, data cleaning, data export for statistical analysis, and event adjudication. Clinically relevant laboratory assessments will be conducted in real-time, while those included in the exploratory list will be analysed in a later time, from frozen serum or plasma. Data from the primary and secondary endpoints will be included in REDCap in the appropriate time points. The independent Data Monitoring Committee (DMC) will have full access to the study data .

The follow-up data for this trial are collected until day 29. A one-year follow-up study of the hospitalized patients enrolled in all Coalition trials will keep monitoring those patients enrolled in this study accepting to participate. Patients who die within this period will be censored at the time of death, and those still hospitalized will be followed on the study until discharge before enrolling in the long-term study. Contacts by telephone and other means will be used to ensure the highest possible retention rate during follow-up. We anticipate a high retention rate up to D29, considering that most of the patients will still be recovering from their recent viral pneumonia. Adherence will be monitored by reconciling protocol specified data collection and the data entry into REDCap.

### Statistical analysis

#### Sample size

Considering an ordinal seven stages outcome with probabilities of 30%, 20%, 8%, 8%, 4%, 15%, 15%, respectively for stages 1 to 7, under the model of proportional odds ratios for the accumulated probabilities for the outcome levels, a sample of 75 cases per arm (150 cases) has an 80% power to detect an odds ratio of 0.44, with a 5% significance level.

### Statistical methods

The main analysis will follow the intention-to-treat principle. The primary endpoint will be assessed by ordinal logistic regression assuming proportional odds ratios adjusted for the stratification variables (age and gender). Logistic regression models will assess binary cumulated outcomes. If odds proportionality does not hold in the final analysis, the primary outcome will be changed to a binomial endpoint collapsing categories 1 to 5 and 6 to 7 (alive versus dead or on mechanical ventilation). Secondary outcomes will be evaluated by generalized linear regressions using appropriate distributions. All models will be adjusted for age and the results will be presented with their 95% confidence intervals for the effect measures. Subgroup analyses will be presented in forest plots evaluated with interaction terms of group and the following variables: age (< or > 60 years), sex, types of comorbidities (cardiovascular, pulmonary, hepatic, renal, obesity, high blood pressure, cancer, diabetes), and inflammatory markers increased at the time of the study entry. Analyses will be performed with R software.^(29)^

### Monitoring

An interim analysis will be performed when 50% of the planned accrual is reached (n = 75). The interim analysis will be performed by an independent DMC who will analyze efficacy and safety data, recruitment rate, adherence to the protocol, data quality, and follow-up losses. These data will be provided by the study coordinating site to the committee via a report. The Lan DeMets method and O’Brien Fleming thresholds will be applied as pre-defined criteria for study interruption. If the committee recommends continued accrual, the study will go onto completion (n = 150).

### Adverse events

Adverse events are defined as any untoward medical occurrence, including an exacerbation of a pre-existing condition, in a subject during a clinical trial. The event does not necessarily have to have a causal relationship with the treatment.

A serious AE is defined as any AE that results in death, offers immediate risk to life, results in persistent or significant disability, requires or prolongs the patient’s hospitalization, or if it is a major medical event that based on proper medical judgment could threaten the patient’s life or could require medical or surgical intervention to prevent one of the other results listed above. Adverse events classified as serious will be notified within 24 hours to the coordinating site.

The AEs will be graded according to the most recent version of the Common Terminology Criteria for Adverse Events (CTCAE). Monthly monitoring will evaluate the integrity of the screening, inclusion/criteria, and data reporting from the sites for adequacy and consistency. Every 15 days, a statistical methodology will be applied to look at inconsistency and data errors. 

Given the biologic characteristics of tocilizumab, the following AEs will be of specific interest and will be captured:

- Secondary infections.
- Anemia.
- Liver function test abnormalities.
- Diverticulitis.
- Herpes zoster.
- Headache.
- Hemorrhage.
- Thromboembolic events.
- Serious infusion-related toxicities.

### Ethics and dissemination

The TOCIBRAS study was approved by local and central (national) ethical committees following current national and international guidelines/directives. The National Committee in Research and Ethics (*Comissão Nacional de Ética em Pesquisa* - Conep) is the entity in Brazil regulating ethical standards for clinical research. TOCIBRAS was developed and will be conducted under the normative set forth by Conep, which adopts international ethical standards in clinical research. Each participating center had the study approved at their institutional review boards before initiating protocol enrolment. A first amendment was submitted to both central and local ethical committees, dealing with issues that were not clear in the original version of the protocol. This amendment did not change the primary or secondary endpoints, the study design, statistics, safety/efficacy monitoring, Informed Consent, exclusion criteria, or interim analysis. The inclusion criteria were not unchanged, except for the limit for the time of intubation up to 24 hours before the inclusion, as the benefit of an anti-inflammatory strategy, including tocilizumab, is more likely to be seen before a fully established SIRS.

Delegated investigators will obtain Informed Consent in this study. This trial only includes adult subjects; thus no rules for minor-aged patients’ consent are considere d. Once the Informed Consent is signed, it is safeguarded by the center’s research personnel. When a written Informed Consent cannot be provided due to the clinical status or other logistical impediments, a verifiable audio consent will be temporarily permitted for study inclusion. The process of audio recording is applied according to Conep guidelines for COVID-19 trials, given the eventual inability to timely obtain a written Informed Consent given the pace of the pandemic and the significant restrictions for visitations and companion stays in the regular wards or the intensive care units treating for COVID-19 patients. Once the clinical circumstances allow, written Informed Consent will be signed by the patient or next of kin and added to the patient’s records. The physician will discuss the risks, benefits, and caveats in participating in the trial and answer all questions. This conversation will be documented in the medical record by the delegated physician, which will reflect the patient’s knowledge about the study and consent. 

The TOCIBRAS is an independent investigator-initiated trial funded by the COALITION COVID-19 Brazil. The exploratory laboratory analysis will be conducted and funded by Fleury Laboratory in São Paulo. A donation from *Instituto Votorantim* was kindly provided for the purchase of tocilizumab for this study. 

The data derived from this trial will be published regardless of the results. The dataset will be analyzed independently by statisticians not involved with the teams involved with data entry and patient care in the participating centers. The publication policy will follow that of the Coalition members. The publication venues will include medical meetings/conferences and submission to peer review journals. Depending on the results, preliminary results could be disclosed via a press release if it is determined that it is in the public’s best interest. The investigators in TOCIBRAS will write the manuscript, which will be approved by all authors before any submissions. 

## CONCLUSION

The result of this trial will shed light on the potential role of an interleukin blocking strategy in patients with moderate to severe COVID-19. Apart from the many other anti-cytokine approaches being investigated in COVID-19, our study selects patients who manifest an ongoing systemic inflammatory response, as evidenced by serum inflammatory biomarkers, which could make this strategy more effective. We hypothesize that tocilizumab could be more active in the earlier stages of the inflammatory response (which tends to occur in the second week of the infection) before it becomes fully established with multiorgan compromise. This approach differs, for example, from antiviral strategies where the more significant benefit is anticipated in the earlier stages of the infection (first week). Therefore the study was designed to detect a difference of tocilizumab plus standard of care versus standard of care in this more select ‘window’ in the disease course in moderate to severe COVID-19. As with most COVID-19 studies, assumptions had to be made in regards to anticipated treatment differences given the scarcity and inconsistency in the best available data available at the time of protocol development in regards to outcomes of patients with COVID-19. Nevertheless, we included a robust design and several secondary clinical and laboratory exploratory endpoints, which will allow us to detect if there is an activity of tocilizumab in this patient population. Our work will complement all the anti-inflammatory and other approaches being developed to mitigate the consequences of COVID-19.

### Strengths and limitations of this study

- The randomized controlled study design permits a more definitive conclusion regarding the activity of tocilizumab in COVID-19.
- The well-defined patient population with a higher propensity to develop inflammatory complications of COVID-19 may define the subset of patients in which a potent anti-inflammatory approach is warranted.
- The 7-point ordinal scale is a robust endpoint for COVID-19 trials.
- The study is not blinded which presents a weakness of the study design.
- The total n is relatively small for a controlled study with more limited statistical power.

## Appendix 1 SPIRIT 2013 checklist

**Table:**

Section/item	Item	Description	Page
**Administrative information**			
Title	1	Descriptive title identifying the study design, population, interventions, and, if applicable, trial acronym	1
Trial registration	2a	Trial identifier and registry name. If not yet registered, name of intended registry	1,2
	2b	All items from the World Health Organization Trial Registration Data Set	1
Protocol version	3	Date and version identifier	
Funding	4	Sources and types of financial, material, and other support	11
Roles and responsibilities	5a	Names, affiliations, and roles of protocol contributors	1
	5b	Name and contact information for the trial sponsor	1, 11
	5c	Role of study sponsor and funders, if any, in study design; collection, management, analysis, and interpretation of data; writing of the report; and the decision to submit the report for publication, including whether they will have ultimate authority over any of these activities	4,11, 16
	5d	Composition, roles, and responsibilities of the coordinating centre, steering committee, endpoint adjudication committee, data management team, and other individuals or groups overseeing the trial, if applicable (see Item 21a for data monitoring committee)	16
**Introduction**			
Background and rationale	6a	Description of research question and justification for undertaking the trial, including summary of relevant studies (published and unpublished) examining benefits and harms for each intervention	3,4
	6b	Explanation for choice of comparators	4
Objectives	7	Specific objectives or hypotheses	5
Trial design	8	Description of trial design including type of trial (eg, parallel-group, crossover, factorial, single group), allocation ratio, and framework (eg, superiority, equivalence, noninferiority, exploratory)	4,6
**Methods: Participants, interventions, and outcomes**			
Study setting	9	Description of study settings (eg, community clinic, academic hospital) and list of countries where data will be collected. Reference to where list of study sites can be obtained	4
Eligibility criteria	10	Inclusion and exclusion criteria for participants. If applicable, eligibility criteria for study centers and individuals who will perform the interventions (eg, surgeons, psychotherapists)	4,5
Interventions	11a	Interventions for each group with sufficient detail to allow replication, including how and when they will be administered	6,7
	11b	Criteria for discontinuing or modifying allocated interventions for a given trial participant (eg, drug dose change in response to harms, participant request, or improving/worsening disease)	NA
	11c	Strategies to improve adherence to intervention protocols, and any procedures for monitoring adherence (eg, drug tablet return, laboratory tests)	6,7
	11d	Relevant concomitant care and interventions that are permitted or prohibited during the trial	6,7
Outcomes	12	Primary, secondary, and other outcomes, including the specific measurement variable (eg, systolic blood pressure), analysis metric (eg, change from baseline, final value, time to event), method of aggregation (eg, median, proportion), and time point for each outcome. Explanation of the clinical relevance of chosen efficacy and harm outcomes is strongly recommended	5,6
Participant timeline	13	Time-schedule of enrolment, interventions (including any run-ins and washouts), assessments, and visits for participants. A schematic diagram is highly recommended	15
Sample size	14	Estimated number of participants needed to achieve study objectives and how it was determined, including clinical and statistical assumptions supporting any sample size calculations	8
Recruitment	15	Strategies for achieving adequate participant enrolment to reach target sample size	7
**Methods: Assignment of interventions (for controlled trials)**			
Allocation:			
Sequence generation	16a	Method of generating the allocation sequence (eg, computer-generated random numbers), and list of any factors for stratification. To reduce predictability of a random sequence, details of any planned restriction (eg, blocking) should be provided in a separate document that is unavailable to those who enrol participants or assign interventions	7
Allocation concealment mechanism	16b	Mechanism of implementing the allocation sequence (eg, central telephone; sequentially numbered, opaque, sealed envelopes), describing any steps to conceal the sequence until interventions are assigned	7
Implementation	16c	Who will generate the allocation sequence, who will enroll participants, and who will assign participants to interventions	7
Blinding (masking)	17a	Who will be blinded after assignment to interventions (eg, trial participants, care providers, outcome assessors, data analysts), and how	7
	17b	If blinded, circumstances under which unblinding is permissible, and procedure for revealing a participant's allocated intervention during the trial	7
**Methods: Data collection, management, and analysis**			
Data collection methods	18a	Plans for assessment and collection of outcome, baseline, and other trial data, including any related processes to promote data quality (eg, duplicate measurements, training of assessors) and a description of study instruments (eg, questionnaires, laboratory tests) along with their reliability and validity, if known. Reference to where data collection forms can be found, if not in the protocol	5,6
	18b	Plans to promote participant retention and complete follow-up, including list of any outcome data to be collected for participants who discontinue or deviate from intervention protocols	NA
Data management	19	Plans for data entry, coding, security, and storage, including any related processes to promote data quality (eg, double data entry; range checks for data values). Reference to where details of data management procedures can be found, if not in the protocol	7,8
Statistical methods	20a	Statistical methods for analyzing primary and secondary outcomes. Reference to where other details of the statistical analysis plan can be found, if not in the protocol	8
	20b	Methods for any additional analyses (eg, subgroup and adjusted analyses)	NA
	20c	Definition of analysis population relating to protocol non-adherence (eg, as randomized analysis), and any statistical methods to handle missing data (eg, multiple imputations)	NA
**Methods: monitoring**			
Data monitoring	21a	Composition of data monitoring committee (DMC); summary of its role and reporting structure; statement of whether it is independent of the sponsor and competing interests; and reference to where further details about its charter can be found, if not in the protocol. Alternatively, an explanation of why a DMC is not needed	8,9
	21b	Description of any interim analyses and stopping guidelines, including who will have access to these interim results and make the final decision to terminate the trial	9
Harms	22	Plans for collecting, assessing, reporting, and managing solicited and spontaneously reported adverse events and other unintended effects of trial interventions or trial conduct	9
Auditing	23	Frequency and procedures for auditing trial conduct, if any, and whether the process will be independent of investigators and the sponsor	8
**Ethics and dissemination**			
Research ethics approval	24	Plans for seeking research ethics committee/institutional review board (REC/IRB) approval	2,10
Protocol amendments	25	Plans for communicating important protocol modifications (eg, changes to eligibility criteria, outcomes, analyses) to relevant parties (eg, investigators, REC/IRBs, trial participants, trial registries, journals, regulators)	10
Consent or assent	26a	Who will obtain informed consent or assent from potential trial participants or authorized surrogates, and how (see item 32)	10
	26b	Additional consent provisions for collection and use of participant data and biological specimens in ancillary studies, if applicable	NA
Confidentiality	27	How personal information about potential and enrolled participants will be collected, shared, and maintained to protect confidentiality before, during, and after the trial	10
Declaration of interests	28	Financial and other competing interests for principal investigators for the overall trial and each study site	11
Access to data	29	Statement of who will have access to the final trial dataset, and disclosure of contractual agreements that limit such access for investigators	NA
Ancillary and post-trial care	30	Provisions, if any, for ancillary and post-trial care, and for compensation to those who suffer harm from trial participation	NA
Dissemination policy	31a	Plans for investigators and sponsor to communicate trial results to participants, healthcare professionals, the public, and other relevant groups (eg, via publication, reporting in results databases, or other data-sharing arrangements), including any publication restrictions	NA
	31b	Authorship eligibility guidelines and any intended use of professional writers	11
	31c	Plans, if any, for granting public access to the full protocol, participant-level dataset, and statistical code	NA
**Appendices**			
Informed consent materials	32	Model consent form and other related documentation provided to participants and authorized surrogates	NA
Biological specimens	33	Plans for collection, laboratory evaluation, and storage of biological specimens for genetic or molecular analysis in the current trial and for future use in ancillary studies, if applicable	NA

NA - not applicable; DMC - data monitoring committee; REC/IRB - research ethics committee/institutional review board.

## Appendix 2 Steering committee

**COALITION COVID-19 Brazil VI Investigators**

Viviane Cordeiro Veiga, Phillip Scheinberg, Danielle Leão Cordeiro de Farias, João Prats, Alexandre Biasi Cavalcanti, Flávia Ribeiro Machado, Regis Goulart Rosa, Otávio Berwanger, Luciano César Pontes de Azevedo, Renato Delascio Lopes, Álvaro Avezum, Leticia Kawano-Dourado, Claudio Galvão for the COALITION COVID-19 Brasil VI Investigators.

## Appendix 3 Exploratory laboratory testing

This appendix includes the details in the methodology of exploratory laboratory testing as part of the secondary endpoints. As it relates to interleukins testing, the measurement of the cytokines IL-6, TNFα, IL-10, as well as the IL-2 receptor (CD25), will be performed by capture ELISA system. Briefly, the serum samples will be incubated in an appropriate dilution in polystyrene plates pre-coated as monoclonal antibodies against the cytokine of interest for 30 minutes. After washing, there will be incubation with peroxidase-labelled monoclonal antibody for 30 minutes. After a new wash, there will be incubation with 3,3 ‘, 5,5’-tetramethylbenzidine (TMB) and hydrogen peroxide (H2O2). After 10 minutes, the reaction will be stopped by adding 1N H2SO4 and each well will be evaluated by spectrophotometry at wavelength 450nm.

As it relates to the flow cytometric studies, all samples will be collected in tubes containing K3 EDTA as anticoagulant. Cells in suspension (2x10^6^ cells in 100 µL per tube) from the peripheral blood samples will be stained with monoclonal antibodies (MAb) directed against cell surface markers using a stain-lyse-and-then-wash, direct immunofluorescence technique. The following panel of 8-color combinations of monoclonal antibodies (MAbs)—fluorescein isothiocyanate (FITC)/phycoerythrin (PE)/peridinin chlorophyll protein (PerCP-Cy5.5)/ PE-cyanine 7 (PE-Cy7)/allophycocyanin (APC)/APC-H7/Brilliant Violet 421 (BV421)/Violet 500 (V500) — will be used in all cases: IgM/CD10/CD20/CD19/IgD/CD38/CD27/CD45, D57/CD26/CD3/CD25/CD279/CD8/CD4/CD45,CD16/CD123/CD34/CD33/CD56/CD3+CD19+CD14/HLA-DR/CD45 and CD8+Ig(K)/CD56+Ig(L)/CD3/CD19+TCR-gamma-delta/CD5/CD38/CD20+CD4/CD38. A tube containing Ig isotype controls for FITC/PE/PerCPCy5.5/PE-Cy7/APC/APC-H7/BV421/V500 will be performed in all cases. The source of MAbs will be as follows: Ig isotype controls, CD3, CD4, CD8, CD5, CD10, CD14, CD16, CD19, CD20, CD25, CD26, CD33, CD34, CD38, CD45, CD56, CD57, CD123, CD279, TCR-gamma-delta, IgD, Ig(K), Ig(L) are from Becton Dickinson Biosciences (BDB), San Jose, CA, USA; HLA-DR are from Biolegend, San Diego, CA, USA; and IgM from Beckman Coulter, Indianapolis, USA. Data acquisition will be performed immediately after completion of sample staining, using a FACSLyric flow cytometer and the FACSuite software (BDB). For each sample, data from at least 3 x 10^5^ events per tube will be acquired. The Infinicyt software (Cytognos, SL, Salamanca, Spain) was used for the analysis of flow cytometry data. Daily instrument quality control was performed using CS&T beads (BDB) to ensure consistent determination of fluorescence intensity during the study.

In relation to the coagulation the following will be performed. All samples will be collected in tubes containing citrate 3,2% as anticoagulant. Tubes will be centrifuged at 2,200 g and plasma was aliquoted and stored at -80? C. For analysis, samples will be thawed at 37? C for 20 minutes. All assays will be performed on ACL TOP 750 analyser (Instrument Laboratories, Bedford, USA) accordingly to standard protocols. The PT will be performed using Hemosil® RecombiPlasntin 2G, PTT and factor 8 assays will be performed using Hemosil® Synthasil and Hemosil® Factor VIII deficient plasma. Factor VIII assay will be performed using a single-point assay (1/20 dilution in buffer). Fibrinogen will be performed using Hemosil® QFA Thrombin (Bovine) reagent by Clauss method. Von Willebrand assay will be performed using Hemosil® VWF: Ag and ristocetin cofactor assay will be performed using Hemosil® VWF:Rco, all immunoturbidimetric tests. All reagents are from Instrument Laboratories (IL, Bedford, USA).
