# Supplementary material for: Rationale and design of the “Tocilizumab in patients with moderate to severe COVID-19: an open-label multicentre randomized controlled” trial (TOCIBRAS)
Source: Rev Bras Ter Intensiva. 2020 Jul-Sep;32(3):337–47. doi: 10.5935/0103-507X.20200060 (PMC7595725; doi:10.5935/0103-507X.20200060)
Supplement: Supplementary file 1 [file rbti-32-03-0337-suppl01.pdf]

Rationale and design of the "Tocilizumab in patients with moderate to severe COVID-19: an open-label multicentre randomized controlled" trial (TOCIBRAS)

Justificativa e delineamento do estudo "Tocilizumabe em pacientes com COVID-19 moderado a grave: estudo aberto, multicêntrico, randomizado, controlado" (TOCIBRAS)

Danielle Leão Cordeiro de Farias[1], João Prats[1], Alexandre Biasi Cavalcanti[2,3], Regis Goulart Rosa[2,4], Flávia Ribeiro Machado[2], Otávio Berwanger[5], Luciano César Pontes de Azevedo[2,6], Renato Delascio Lopes[7,8], Álvaro Avezum[9], Leticia Kawano-Dourado[3], Lucas Petri Damiani[3], Salomón Soriano Ordinola Rojas[1], Cleyton Zanardo de Oliveira[1], Luis Eduardo Coelho Andrade[10], Alex Freire Sandes[10], Maria Carolina Pintão[10], Claudio Galvão de Castro Júnior[5,11], Phillip Scheinberg[1], Viviane Cordeiro Veiga[1,2] for the COALITION COVID-19 BRAZIL VI

Appendix 1. SPIRIT 2013 checklist

Section/item	Item	Description	Page
**Administrative information**			
Title	1	Descriptive title identifying the study design, population, interventions, and, if applicable, trial acronym	1
Trial registration	2a	Trial identifier and registry name. If not yet registered, name of intended registry	1,2
	2b	All items from the World Health Organization Trial Registration Data Set	1
Protocol version	3	Date and version identifier	
Funding	4	Sources and types of financial, material, and other support	11
Roles and responsibilities	5a	Names, affiliations, and roles of protocol contributors	1
	5b	Name and contact information for the trial sponsor	1, 11
	5c	Role of study sponsor and funders, if any, in study design; collection, management, analysis, and interpretation of data; writing of the report; and the decision to submit the report for publication, including whether they will have ultimate authority over any of these activities	4,11, 16
	5d	Composition, roles, and responsibilities of the coordinating centre, steering committee, endpoint adjudication committee, data management team, and other individuals or groups overseeing the trial, if applicable (see Item 21a for data monitoring committee)	16
**Introduction**			
Background and rationale	6a	Description of research question and justification for undertaking the trial, including summary of relevant studies (published and unpublished) examining benefits and harms for each intervention	3,4
	6b	Explanation for choice of comparators	4
Objectives	7	Specific objectives or hypotheses	5
Trial design	8	Description of trial design including type of trial (eg, parallel-group, crossover, factorial, single group), allocation ratio, and framework (eg, superiority, equivalence, noninferiority, exploratory)	4,6
**Methods: Participants, interventions, and outcomes**			
Study setting	9	Description of study settings (eg, community clinic, academic hospital) and list of countries where data will be collected. Reference to where list of study sites can be obtained	4
Eligibility criteria	10	Inclusion and exclusion criteria for participants. If applicable, eligibility criteria for study centers and individuals who will perform the interventions (eg, surgeons, psychotherapists)	4,5
Interventions	11a	Interventions for each group with sufficient detail to allow replication, including how and when they will be administered	6,7
	11b	Criteria for discontinuing or modifying allocated interventions for a given trial participant (eg, drug dose change in response to harms, participant request, or improving/worsening disease)	NA
	11c	Strategies to improve adherence to intervention protocols, and any procedures for monitoring adherence (eg, drug tablet return, laboratory tests)	6,7
	11d	Relevant concomitant care and interventions that are permitted or prohibited during the trial	6,7
Outcomes	12	Primary, secondary, and other outcomes, including the specific measurement variable (eg, systolic blood pressure), analysis metric (eg, change from baseline, final value, time to event), method of aggregation (eg, median, proportion), and time point for each outcome. Explanation of the clinical relevance of chosen efficacy and harm outcomes is strongly recommended	5,6
Participant timeline	13	Time-schedule of enrolment, interventions (including any run-ins and washouts), assessments, and visits for participants. A schematic diagram is highly recommended	15
Sample size	14	Estimated number of participants needed to achieve study objectives and how it was determined, including clinical and statistical assumptions supporting any sample size calculations	8
Recruitment	15	Strategies for achieving adequate participant enrolment to reach target sample size	7

**Methods: Assignment of interventions (for controlled trials)**			
Allocation:			
Sequence generation	16a	Method of generating the allocation sequence (eg, computer-generated random numbers), and list of any factors for stratification. To reduce predictability of a random sequence, details of any planned restriction (eg, blocking) should be provided in a separate document that is unavailable to those who enrol participants or assign interventions	7
Allocation concealment mechanism	16b	Mechanism of implementing the allocation sequence (eg, central telephone; sequentially numbered, opaque, sealed envelopes), describing any steps to conceal the sequence until interventions are assigned	7
Implementation	16c	Who will generate the allocation sequence, who will enroll participants, and who will assign participants to interventions	7
Blinding (masking)	17a	Who will be blinded after assignment to interventions (eg, trial participants, care providers, outcome assessors, data analysts), and how	7
	17b	If blinded, circumstances under which unblinding is permissible, and procedure for revealing a participant's allocated intervention during the trial	7
**Methods: Data collection, management, and analysis**			
Data collection methods	18a	Plans for assessment and collection of outcome, baseline, and other trial data, including any related processes to promote data quality (eg, duplicate measurements, training of assessors) and a description of study instruments (eg, questionnaires, laboratory tests) along with their reliability and validity, if known. Reference to where data collection forms can be found, if not in the protocol	5,6
	18b	Plans to promote participant retention and complete follow-up, including list of any outcome data to be collected for participants who discontinue or deviate from intervention protocols	NA
Data management	19	Plans for data entry, coding, security, and storage, including any related processes to promote data quality (eg, double data entry; range checks for data values). Reference to where details of data management procedures can be found, if not in the protocol	7,8
Statistical methods	20a	Statistical methods for analyzing primary and secondary outcomes. Reference to where other details of the statistical analysis plan can be found, if not in the protocol	8
	20b	Methods for any additional analyses (eg, subgroup and adjusted analyses)	NA
	20c	Definition of analysis population relating to protocol non-adherence (eg, as randomized analysis), and any statistical methods to handle missing data (eg, multiple imputations)	NA
**Methods: Monitoring**			
Data monitoring	21a	Composition of data monitoring committee (DMC); summary of its role and reporting structure; statement of whether it is independent of the sponsor and competing interests; and reference to where further details about its charter can be found, if not in the protocol. Alternatively, an explanation of why a DMC is not needed	8,9
	21b	Description of any interim analyses and stopping guidelines, including who will have access to these interim results and make the final decision to terminate the trial	9
Harms	22	Plans for collecting, assessing, reporting, and managing solicited and spontaneously reported adverse events and other unintended effects of trial interventions or trial conduct	9
Auditing	23	Frequency and procedures for auditing trial conduct, if any, and whether the process will be independent of investigators and the sponsor	8
**Ethics and dissemination**			
Research ethics approval	24	Plans for seeking research ethics committee/institutional review board (REC/IRB) approval	2,10
Protocol amendments	25	Plans for communicating important protocol modifications (eg, changes to eligibility criteria, outcomes, analyses) to relevant parties (eg, investigators, REC/IRBs, trial participants, trial registries, journals, regulators)	10
Consent or assent	26a	Who will obtain informed consent or assent from potential trial participants or authorized surrogates, and how (see item 32)	10
	26b	Additional consent provisions for collection and use of participant data and biological specimens in ancillary studies, if applicable	NA
Confidentiality	27	How personal information about potential and enrolled participants will be collected, shared, and maintained to protect confidentiality before, during, and after the trial	10
Declaration of interests	28	Financial and other competing interests for principal investigators for the overall trial and each study site	11
Access to data	29	Statement of who will have access to the final trial dataset, and disclosure of contractual agreements that limit such access for investigators	NA
Ancillary and post-trial care	30	Provisions, if any, for ancillary and post-trial care, and for compensation to those who suffer harm from trial participation	NA
Dissemination policy	31a	Plans for investigators and sponsor to communicate trial results to participants, healthcare professionals, the public, and other relevant groups (eg, via publication, reporting in results databases, or other data-sharing arrangements), including any publication restrictions	NA
	31b	Authorship eligibility guidelines and any intended use of professional writers	11
	31c	Plans, if any, for granting public access to the full protocol, participant-level dataset, and statistical code	NA
**Appendices**			
Informed consent materials	32	Model consent form and other related documentation provided to participants and authorized surrogates	NA
Biological specimens	33	Plans for collection, laboratory evaluation, and storage of biological specimens for genetic or molecular analysis in the current trial and for future use in ancillary studies, if applicable	NA

NA - not applicable; DMC - data monitoring committee; REC/IRB - research ethics committee/institutional review board.

APPENDIX 2 - STEERING COMMITTEE

COALITION COVID-19 Brazil VI Investigators

Viviane Cordeiro Veiga, Phillip Scheinberg, Danielle Leão Cordeiro de Farias, João Prats, Alexandre Biasi Cavalcanti, Flávia Ribeiro Machado, Regis Goulart Rosa, Otávio Berwanger, Luciano César Pontes de Azevedo, Renato Delascio Lopes, Álvaro Avezum, Leticia Kawano-Dourado, Claudio Galvão for the COALITION COVID-19 Brasil VI Investigators.

APPENDIX 3 - EXPLORATORY LABORATORY TESTING

This appendix includes the details in the methodology of exploratory laboratory testing as part of the secondary endpoints. As it relates to interleukins testing, the measurement of the cytokines IL-6, TNFα, IL-10, as well as the IL-2 receptor (CD25), will be performed by capture ELISA system. Briefly, the serum samples will be incubated in an appropriate dilution in polystyrene plates pre-coated as monoclonal antibodies against the cytokine of interest for 30 minutes. After washing, there will be incubation with peroxidase-labelled monoclonal antibody for 30 minutes. After a new wash, there will be incubation with 3,3 ', 5,5'-tetramethylbenzidine (TMB) and hydrogen peroxide (H2O2). After 10 minutes, the reaction will be stopped by adding 1N H2SO4 and each well will be evaluated by spectrophotometry at wavelength 450nm.

As it relates to the flow cytometric studies, all samples will be collected in tubes containing K3 EDTA as anticoagulant. Cells in suspension ($2x10^6$ cells in 100 μL per tube) from the peripheral blood samples will be stained with monoclonal antibodies (MAb) directed against cell surface markers using a stain-lyse-and-then-wash, direct immunofluorescence technique. The following panel of 8-color combinations of monoclonal antibodies (MAbs)—fluorescein isothiocyanate (FITC)/phycoerythrin (PE)/peridinin chlorophyll protein (PerCP-Cy5.5)/ PE–cyanine 7 (PE-Cy7)/allophycocyanin (APC)/APC-H7/Brilliant Violet 421 (BV421)/Violet 500 (V500) — will be used in all cases: IgM/CD10/CD20/CD19/IgD/CD38/CD27/CD45, D57/CD26/CD3/CD25/CD279/CD8/CD4/CD45,CD16/CD123/CD34/CD33/CD56/CD3+CD19+CD14/HLA-DR/CD45 and CD8+Ig(K)/CD56+Ig(L)/CD3/CD19+TCR-gamma-delta/CD5/CD38/CD20+CD4/CD38. A tube containing Ig isotype controls for FITC/PE/PerCPCy5.5/PE-Cy7/APC/APC-H7/BV421/V500 will be performed in all cases. The source of MAbs will be as follows: Ig isotype controls, CD3, CD4, CD8, CD5, CD10, CD14, CD16, CD19, CD20, CD25, CD26, CD33, CD34, CD38, CD45, CD56, CD57, CD123, CD279, TCR-gamma-delta, IgD, Ig(K), Ig(L) are from Becton Dickinson Biosciences (BDB), San Jose, CA, USA; HLA-DR are from Biolegend, San Diego, CA, USA; and IgM from Beckman Coulter, Indianapolis, USA. Data acquisition will be performed immediately after completion of sample staining, using a FACSLyric flow cytometer and the FACSuite software (BDB). For each sample, data from at least 3×10^5 events per tube will be acquired. The Infinicyt software (Cytognos, SL, Salamanca, Spain) was used for the analysis of flow cytometry data. Daily instrument quality control was performed using CS&T beads (BDB) to ensure consistent determination of fluorescence intensity during the study.

In relation to the coagulation the following will be performed. All samples will be collected in tubes containing citrate 3,2% as anticoagulant. Tubes will be centrifuged at 2,200 g and plasma was aliquoted and stored at -80° C. For analysis, samples will be thawed at 37° C for 20 minutes. All assays will be performed on ACL TOP 750 analyser (Instrument Laboratories, Bedford, USA) accordingly to standard protocols. The PT will be performed using Hemosil® Recombi-Plasntin 2G, PTT and factor 8 assays will be performed using Hemosil® Synthasil and Hemosil® Factor VIII deficient plasma. Factor VIII assay will be performed using a single-point assay (1/20 dilution in buffer). Fibrinogen will be performed using Hemosil® QFA Thrombin (Bovine) reagent by Clauss method. Von Willebrand assay will be performed using Hemosil® VWF: Ag and ristocetin cofactor assay will be performed using Hemosil® VWF:Rco, all immunoturbidimetric tests. All reagents are from Instrument Laboratories (IL, Bedford, USA).
